# Psychometric properties of the 10-item ruminative response scale in Chinese university students

**DOI:** 10.1186/s12888-017-1318-y

**Published:** 2017-04-28

**Authors:** Xiaoxia Lei, Mingtian Zhong, Ying Liu, Chang Xi, Yu Ling, Xiongzhao Zhu, Shuqiao Yao, Jinyao Yi

**Affiliations:** 1 Medical Psychological Center, Second Xiangya Hospital of Central South University, #139 Renmin Road, Changsha, 410011 China; 2 Center for Studies of Psychological Application, School of Psychology, South China Normal University, Guangzhou, 510631 People’s Republic of China; 3 Education Institute, Hunan Agricultural University, Changsha, 410128 People’s Republic of China; 4 Medical Psychological Institute, Central South University, Changsha, 410011 People’s Republic of China

**Keywords:** Depression, Rumination, RRS-10, Reliability, Validity, Measurement equivalence, Gender difference

## Abstract

**Background:**

Rumination increases vulnerability to depression, exacerbates and perpetuates negative moods. This study was aimed to examine the psychometric properties of the Chinese version of the 10-item Ruminative Response Scale (RRS-10) in a large undergraduate sample.

**Methods:**

A sample of 5,236 university students finished the RRS and the Center for Epidemiological Studies Depression Scale (CES-D). Confirmatory Factor Analysis (CFA) was performed to examine the two-factor structure and the measurement equivalence of the RRS-10 across gender. The internal consistency, test-retest reliability, correlations among RRS, RRS-10 and CES-D were also explored. In addition, gender difference on rumination and the relationship between rumination and depression were further investigated.

**Results:**

The two-factor model of RRS-10 fit the data reasonably and had acceptable internal consistency and test-retest reliability in Chinese undergraduates sample. And the measurement equivalence of the RRS-10 was acceptable across gender in Chinese university students. Findings in respect of latent means and manifest means revealed non-significant gender difference in RRS-10. Besides, participants with high-level rumination had more depressive symptoms than those with low-level rumination.

**Conclusions:**

The Chinese version of the RRS-10 showed good psychometric properties and was measurement invariant across gender in undergraduates.

**Electronic supplementary material:**

The online version of this article (doi:10.1186/s12888-017-1318-y) contains supplementary material, which is available to authorized users.

## Background

In 1987, Susan Nolen-Hoeksema first put forward the notable ruminative response style theory of depression. In her theory, ruminative responses to depression could be defined as behaviors and thoughts which focus one’s attention on depressive symptoms. Two key points were extracted from her theory. Firstly, ruminative responses aggravate and prolong depressive episodes. Secondly, women are more likely to amplify their depressed moods by ruminating about the possible causes of their depressed states than men [1].

Plenty of studies supported the first point. Nolen and Morrow confirmed that the degree of rumination had a great impact on remediation of depressive mood, with greater rumination leading to lesser remediation of depression [2]. Based on one research in people suffering from an earthquake, Nolen and Morrow also found subjects who had a ruminative response style before the earthquake were more likely to be depressed after the earthquake than those with less rumination [3]. In a subsequent empirical study, Nolen demonstrated that ruminative responses prolong depression because the depressed mood would have negative effect on thinking and problem-solving behavior in participants with depressive disorder [4]. A Nolen’s in-depth research suggested that the more ruminative responses people engaged in, the longer their periods of depressed mood [5]. Other relevant researches also supported Nolen’s findings, recently, a meta analysis concerning the relationship between rumination and depression found that rumination had a significantly positive correlation with depression, and could predict the onset and development of depression [6].

In order to assess the severity of depressive rumination, the 22-item Ruminative Response Scale (RRS) was developed, which asks subjects to report what they generally do when they feel sad, down, or depressed [3]. Other depressive rumination related scales were also developed for different age group or different content, such as the Children’s RRS, Adapted RRS, and rumination sub-scale of Cognitive Emotion Regulation Questionnaire [7]. Among them, the RRS has emerged as the most frequently used self-report measure of depressive rumination in both research and clinical practice for its wide target population and stable reliability and validity [8]. However, following its widespread use, some researchers questioned whether the observed relationship between rumination and depression was due to similar item content of RRS and depression related scales [9–11]. Given that some items on the RRS appear to overlap with items on measures of depressive symptoms, Treynor et al. removed 12 depression-related items from the RRS, and eventually developed a simplified scale called the 10-item Ruminative Response Scale (RRS-10, Treynor et.al., 2003). The RRS-10 includes two sub-scales (Brooding and Reflection), and each has five items. Brooding rumination refers to “mood pondering” (e.g., “I think why can’t I handle things better?”), whereas reflective rumination describes thoughtful and non-emotional reassessment of past and present events, feelings, and behaviors (e.g., “write down what you are thinking, and analyze it”) [12]. Using the simplified scale and eliminating the influence of the depression factor of RRS, researchers did find the scores of RRS-10 were better demonstrated the relationship between rumination and depression [13–15]. The RRS-10 had been reported to have good psychometric properties in both English-speaking and non-English speaking countries, e.g. American, Spanish, Dutch, Korean, and Thailand [14, 16–19]. However, no relevant researches of RRS-10 were reported in Chinese. Thus, due to different cultural background, it is necessary for us to examine the psychometric properties of RRS-10 in Chinese sample, which can provide a credible and effective measuring tool for rumination related research.

Existing laboratory and field studies found that women, when depressed, tend to ruminate more about the possible causes of their mood than men [20–23], which supported the second key point of Nolen’s response styles theory that there exists significant difference between women and men on rumination. Noteworthily, in these studies, researchers all used raw scores of RRS-10 to compare the level of rumination between male and female, which ignored an important issue. When comparing the discrepancy between different groups, a crucial precondition is that all items of the scale must imply equivalent meaning between groups [24, 25]. Therefore, one may question whether the significant gender difference on rumination found in previous studies came from the non-equivalence measuring tool itself or from the real gender difference. Therefore, if we want to compare the true gender difference on rumination, it is necessary to examine the measurement equivalence of the rumination scale across gender firstly [26, 27]. In addition, the latent mean comparison, which measures the latent structure of the scale, ensures that the set of common factors are entirely responsible for all observed differences in the means of the measured variables [27]. In other words, only when the latent structure were equivalent across different groups, the observed variable means (also called manifest variable means) can truly predict the true difference among different groups. Thus, the latent mean differences of gender on RRS-10 factors should be examined before checking the manifest variable mean differences across gender.

Therefore, using a large sample of Chinese university students, this study was aimed to test the psychometric properties of RRS-10, which included three respects: (1)Whether RRS-10’s two factor structure fits the data well; (2)Whether there exists measurement equivalence across gender in this two factor model; (3)Whether the RRS-10 has a good reliability and validity in Chinese sample. The gender difference on rumination was also examined. Meanwhile, the relationship between depression and rumination was further investigated.

## Methods

### Subjects

Subjects were recruited from two Chinese universities in Hunan Province and finished the scales in classroom anonymously. A total of 8022 university students completed the survey and 184 (2.3%) subjects were excluded for data missing in some items, so 7838 subjects with full data (5220 male, 2618 female) were left. To make the number of boys equal to girls, we sampled 2618 subjects from male dataset randomly by the function of “Random sample of cases” in SPSS.20.0. Finally, the sample used in this study consisted of 2618 men (50%), with a mean age of 23.96 (SD = 0.82) and 2618 women (50%), with a mean age of 23.92 (SD = 0.97). The whole sample aged from 22 to 26 years old, with a mean age of 23.94 (SD = 0.90).

A small group of 73 university students from another University finished the RRS-10 twice with a 2-week interval to estimate test-retest reliability. The sample consisted of 44 men with a mean age of 25.32 (SD =2.38) and 29 women with a mean age of 21.33 (SD =0.96). The whole sample aged from 21 to 27 years old, with a mean age of 23.8 (SD = 2.76).

This protocol was approved by the ethics committee of Central South University. Every participant signed an informed consent form at enrollment.

## Instruments

### Ruminative response scale and the 10-item ruminative response scale 

The ruminative response scale (RRS), a self-report measure of describing one’s responses to depressed mood, consists of 22 items and three factors (Depression, Brooding, and Reflection). Each item are rated on a 4-point Likert scale ranging from 1 (never) to 4 (always). The total score ranges from 22 to 88, with higher scores indicating higher degrees of ruminative symptoms. Nolen et al. had reported acceptable levels of internal consistency [3]. The Chinese version RRS was developed through forward and back translation by separate bilingual translators. Any discrepancy arising in back translation was resolved through cooperation between translators to correct the Chinese version until consensus was achieved. No questionnaire item was removed or altered significantly during translation.

The 10-item ruminative response scale (RRS-10), is a 10-item questionnaire derived from the original 22-item RRS, with the depression factor being removed. In this study, we assessed the full RRS and used ten RRS items to form the RRS-10 scores.

### The center for epidemiologic studies depression scale

The Center for Epidemiologic Studies Depression Scale (CES-D), a laboratory measure of depression, was developed to assess depressive symptoms [28]. The CES-D comprises 20 items, and employs 4-point Likert scales, ranging from “rarely or none of the time” (1 point) to “most or all of the times” (4 points). The total score ranges from 20 to 80, in which a higher score indicates more severe depressive symptoms. The CES-D, which comprises four dimensions (depressed affect, positive affect, somatic complaints, and interpersonal relationships), has shown good reliability and validity in Chinese sample [29]. As a depression related scale, the CES-D can be used to assess convergent validity of the RRS-10 [30, 31], since the conceptualization of rumination in our study was related to depressive mood or depressive symptoms.

## Data analysis

Data analysis was operated on IBM SPSS Statistics 20.0 and AMOS17.0.

Firstly, the Confirmatory Factor Analysis (CFA) method was used to examine the two-factor structure of RRS-10. The maximum likelihood (ML), a common method of estimation within CFA, was adopted in the analysis [32]. To evaluate model fit, we used several fit indexes including the Goodness of Fit Index (GFI), Incremental Fit Index (IFI), Parsimonious Goodness of Fit Index (PGFI), Comparative Fit Index (CFI), the Tucker–Lewis Index (TLI) and Root Mean Square Error of Approximation (RMSEA) with a 90% Confidence Interval (CI). Generally, GFI ≥ 0.90, IFI ≥ 0.90, PGFI ≥ 0.50, CFI ≥ 0.90, TLI ≥ 0.90 and RMSEA ≤ 0.08 can be considered acceptable [33].

Secondly, the Multigroup Confirmatory Factor Analysis (MGCFA) method was applied to the measurement equivalence of RRS-10 across gender. Measurement equivalence tests from low to high included four levels: configural equivalence, weak equivalence, strong equivalence and strict equivalence, which had hierarchical relationships [34]. (1) Configural equivalence (Model 1): an initial analysis with no constraints was tested, with the factor loadings, intercepts of variables and error variances being set free. (2) Weak equivalence or metric equivalence (model 2): constraints of equivalent factor loadings were imposed to make sure whether the RRS-10 had the same structure and meaning across gender, whereas intercepts of variables and error variances were estimated freely. (3) Strong equivalence (model 3): the factor loadings, intercepts of variables were constrained to be equal across gender. (4) Strict equivalence (model 4): the factor loadings, intercepts of variables and error variances were all set to be equal across gender. Posterior models were nested on the former one. Only when the former model was set up, the next model was allowed to be done. Three comparing indexes were chosen: the change in *χ*2 (△*χ*2), the change in degree of freedom (△df) and the change in CFI (△CFI) between two adjacent models. In general, △*χ*2 and △df not reaching statistical significance (*p* >0.05), and △CFI ≤ 0.010 are considered evidence of equivalence [35].

Thirdly, to evaluate the internal consistency, we calculated Cronbach’s alphas (α) and mean inter-item correlation (M_IC_) of RRS-10. Relationships among RRS-10, RRS and CES-D were examined using Pearson’s r coefficient.

Fourthly, the latent mean differences across gender were tested in the structural means model of MGCFA. Male was selected as the reference group, and the latent mean of this group was constrained to 0, while the latent mean of female was estimated freely. The critical ratio (CR) was chosen as the index to evaluate whether the latent means was different across gender [36]. CR for mean was defined as “Dividing the estimate of the mean by the estimate of its standard error gives” in Amos. If the absolute value of CR is < 1.96, the latent means differences across gender has non-statistical significance, otherwise, it does [25]. After comparing the latent means, we calculated the manifest means in total and subscale scores of RRS, RRS-10, and CES-D in different gender groups and used Independent Samples *T* test and Cohen’s *d* effect sizes [37] to indicate the standardized mean difference between male and female.

Finally, to assess the potential relationship between rumination and depression, two groups (high-level rumination and low-level rumination) were selected from the whole sample. Subjects who scored higher than the mean plus one standard deviation of RRS-10’s total scores were defined as the high-level rumination group, while those who scored lower than the mean minus one standard deviation were defined as the low-level rumination group. Then Independent Samples *T* test was used to exam whether there existed statistical significant differences on the CES-D scores between low-level rumination group and high-level group.

## Results

### The goodness of fit indexes for the two-factor model

The fit indexes of CFA were summarized in Table 1. Indexes in all samples met the criterion. In detail, GFI, IFI, TLI, CFI were all > 0.90, the RMSEA values with a 90% confidence interval (CI) were < 0.08, and the PGFI values were > 0.50. Factor loadings and inter-factor correlation were shown in Additional file 1: Appendix 1. These results indicated that the two-factor model proposed by Treynor fitted the data reasonably, and factor loading of most items were within a reasonable range, except for the item 1. Thus, the model could serve as the baseline model for the following measurement equivalence tests.

**Table 1:** Goodness of fit indexes for the two-factor model of RRS-10

	*χ*2	df	GFI	PGFI	IFI	TLI	CFI	RMSEA	RMSEA 90% CI	
									LO90	HI90
Full sample	558.444	30	0.978	0.534	0.943	0.914	0.943	0.058	0.054	0.062
Male sample	253.050	30	0.980	0.535	0.949	0.923	0.948	0.053	0.047	0.059
Female sample	353.470	30	0.973	0.531	0.934	0.901	0.934	0.064	0.058	0.070

*χ*2 Chi-square, *df* degrees of freedom, *GFI* the goodness-of-fit index, *PGFI* parsimonious goodness-of-fit index, *IFI* the incremental fit index, *TLI* he Tucker– Lewis Index, *CFI* Comparative Fit Index, *RMSEA* root-mean-square error of approximation, LO90 and HI90 indicate lower and upper end of the 90% confidence interval of the RMSEA

### Measurement equivalence across gender

The results of measurement equivalence tests across gender were shown in Table 2. The △*χ*2 didn’t reach significant level in all models (*p* >0.05). Small changes of CFI were figured out between two adjacent models (*△CFI* < 0.010). Other fit indexes (IFI, TLI, CFI, RMSEA, GFI, and PGFI) all met the criterion. Results showed that the RRS-10 has good measurement equivalence across gender.

**Table 2:** Fit indexes for measurement equivalence tests of RRS-10

Model	*χ*2	df	IFI	TLI	CFI	RMSEA	GFI	PGFI	model comparison	△CFI	△*χ*2 (△df)	*P* Value	RMSEA 90% CI	
													LO90	HI90
Model 1	606.520	60	0.941	0.911	0.941	0.042	0.977	0.533					0.039	0.045
Model 2	613.650	70	0.941	0.924	0.941	0.039	0.976	0.621	2 vs 1	0.000	7.129(10)	0.713	0.036	0.041
Model 3	614.338	71	0.941	0.925	0.941	0.038	0.976	0.630	3 vs 2	0.001	0.688(1)	0.407	0.034	0.039
Model 4	635.907	85	0.940	0.937	0.940	0.035	0.976	0.754	4 vs 3	0.003	21.569(14)	0.088	0.031	0.036

Model 1 = configural equivalence; Model 2 = metric equivalence;Model 3 = strong equivalence; Model 4 = strict equivalence

### Reliability

As shown in Table 3, Cronbach’s α coefficient was 0.75 for the whole sample and ranged from 0.61 to 0.70 for the two sub-scales, showing acceptable internal consistency in the Chinese undergraduate sample. The M_IC_ ranged from 0.23 to 0.33. Test-retest reliabilities were greater than 0.70, except for the brooding factor in total sample and female sample.

**Table 3:** Cronbach’s alpha, M_IC_ and test-retest reliability of RRS-10

	Total sample			Male sample			Female sample		
	B	R	T	B	R	T	B	R	T
Cronbach’s α	0.61	0.70	0.75	0.60	0.70	0.75	0.62	0.71	0.76
M_IC_	0.23	0.33	0.23	0.23	0.32	0.23	0.24	0.33	0.24
TRT	0.64	0.81	0.76	0.73	0.83	0.77	0.41	0.76	0.72

*B* Brooding subscale, *R* Reflection subscale, *T* Total scale, *M*_*IC*_ mean inter-item correlation

TRT = test-retest reliabilities

### Correlations among RRS-10, RRS and CES-D

Correlations among scales showed that RRS-10 was little correlated with CES-D (0.26), whereas RRS was highly correlated with CES-D (0.75). Among them, the correlation coefficient between the depression factor of RRS and CES-D was the largest (0.96). Subscale intercorrelations showed that the brooding factor was moderately correlated with the reflection factor (0.47), while both the two factors were largely correlated with the RRS-10 (0.83, 0.89, respectively). See details in Table 4.

**Table 4:** Correlations among RRS-10, RRS and CES-D

scale	Depression factor	Brooding factor	Reflection factor	RRS-10	RRS
Brooding factor	0.13^a^				
Reflection factor	0.19^a^	0.47^a^			
RRS-10	0.14^a^	0.83^a^	0.89^a^		
RRS	0.69^a^	0.62^a^	0.76^a^	0.81^a^	
CES-D	0.96^a^	0.14	0.29^a^	0.26^a^	0.75^a^

^a^Correlation is significant at the 0.01 level (2-tailed)

### Latent mean differences of brooding and reflection between male and female

Results of latent mean differences across gender showed that an absolute value of CR as large as 1.742 was figured out in the structural means model of MGCFA for the brooding factor across gender (*p* >0.05). In other words, the mean of brooding factor between male and female was not significantly different at the 0.05 level (two-tailed), neither was the reflecting factor (CR > 1.333, *p* >0.05).

Since there were no significant differences on latent means of brooding and reflection between male and female, the scores of RRS-10 between gender groups could be compared directly.

### Comparison of manifest means of RRS-10, RRS, and CES-D between male and female

As Table 5 showed, there were no significant differences on the total and sub-scales scores of RRS-10 between gender groups. However, there were significant differences on the total score of RRS and the depression factor, as well as the score of CES-D.

**Table 5:** Comparison of RRS-10, RRS, and CES-D between male and female

Scale	Male (*n* = 2618)	Female (*n* = 2618)	Mean difference	*P* value	Cohen’s *d*
Depression	19.81 ± 2.59	21.94 ± 4.22	-2.13	0.00	-0.61
Brooding	10.49 ± 3.37	10.60 ± 2.41	-0.12	0.08	---
Reflection	10.67 ± 2.87	10.76 ± 2.93	-0.09	0.26	---
RRS-10	21.15 ± 4.48	21.36 ± 4.59	-0.21	0.10	---
RRS	40.97 ± 7.02	43.30 ± 5.00	-2.33	0.00	-0.38
CES-D	32.23 ± 6.74	33.76 ± 8.00	-1.53	0.00	-0.21

### Comparison of scores on CES-D between low-level rumination group and high-level group

According to the partition criterion mentioned above, 749 subjects were classified as the low-level rumination group, and 874 subjects were classified as the high-level rumination group. Independent-Samples *T* Test showed that the differences on CES-D scores between low-level rumination group and high-level group were significant, with moderate effect size ranging from -0.46 to -0.65 (Table 6). In addition, the measurem ent equivalence of the RRS-10 was also acceptable across high- and low- level of depressive symptom in a large Chinese undergraduate sample (Additional file 1: Appendix 2).

**Table 6:** Comparison of CES-D and its subscales between low-level rumination group and high-level group

Scale	low-level rumination (*n* = 749)	high-level rumination (*n* = 874)	Mean difference	*P* value	Cohen’s *d*
Depressed Affect	10.48 ± 4.00	12.59 ± 3.52	-2.11	0.00	-0.56
Positive Affect	13.18 ± 2.19	12.00 ± 2.34	1.18	0.00	0.51
Somatic Complaints	10.68 ± 3.40	12.78 ± 3.15	-2.10	0.00	-0.64
Interpersonal	2.76 ± 1.12	3.30 ± 1.22	-0.53	0.00	-0.46
CES-D	30.75 ± 9.62	36.66 ± 8.48	-5.92	0.00	-0.65

## Discussion

In this study, using a large sample of 5236 university students, we focused on the psychometric properties of RRS-10, and explored the gender difference on rumination and relationship between rumination and depression.

Previous researches concerning RRS-10’s factor analysis have shown a good two-factor structure [14, 18]. In the study of Xavier et al., the CFA’s results (GFI = 0.93, CFI = 0.90, TLI = 0.87, RMSEA = 0.11, 90% C.I. = 0.092 to 0.121) showed that the two-factor structure fit the data reasonably [15], as well as in our study. MGCFA’s results showed that the configural equivalence, weak equivalence, strong equivalence and strict equivalence were all tenable between male and female. Namely, the RRS-10 has good measurement equivalence across gender.

There have been six studies which comprehensively reported the internal consistency of RRS-10, with the α coefficients of the brooding factor ranging from 0.62 to 0.86, those of reflection factor ranging from 0.72 to 0.88, and those of the total RRS-10 ranging from 0.80 to 0.86 [3, 15, 16, 18, 19]. Cronbach’s α coefficients in our study were 0.75 for total scale and ranged from 0.61 to 0.70 for two sub-scales, which were little lower than those in previous studies [3, 15, 16, 18, 19]. Noteworthily, there are only 5 items in each factor, and the sample size was large, both of which would bring a lot of uncontrollable factitious factors. Taking these into consideration, our results still showed acceptable internal consistency of the RRS-10 in the Chinese undergraduate sample. The mean inter-item correlations, ranging from 0.23 to 0.33, were all within the optimal range of 0.20–0.40 [38]. The two-week test-retest reliabilities were greater than 0.70, except for the brooding factor in total sample and female sample, which might be due to the small test-retest sample size, after all, there were just 29 female completing the questionnaire.

Correlations among the RRS-10, RRS and CES-D showed that the depression factor of RRS was the highest correlated with CES-D, while the brooding and reflection factor were not. In order to better investigate the relationship between rumination and depression, and eliminate the effect of depression factor, Treynor deleted the depression factor from the RRS for its overlap with depressed related scales [14]. In this study, when the depression items removed, correlations between the total and sub-scales of RRS-10 were both above 0.80, which is consistent with Thanoi’s report [16]. Our results provide further supports for Treynor’s point that the two-factor model of RRS-10 was superior to the three-factor model of RRS.

On the basis of measurement equivalence, the difference on rumination between male and female was evaluated by comparing the latent means and the manifest means of RRS-10. The results of latent and manifest means both demonstrated that there were no significant differences on the brooding and reflecting factors across gender (*p >*0.05), which was consistent with Lee’s, Knowles’ and Hong’s studies [18, 39, 40]. Whereas, several previous studies draw a conclusion that female scored higher in total and sub-scales of RRS, and women seemed to ruminate more about negative mood than men [1, 12, 19, 23]. This inconsistency may be attributed to several aspects as follows. Firstly, some previous studies used RRS, which includes the depression factor. The difference between male and female in previous studies might be due to the depression factor, as our results have shown that there was significant gender difference on depression factor (*p* <0.05, Cohen’s *d* = -0.61) and the whole RRS (*p* <0.05, Cohen’s *d* = -0.38), but not on brooding or reflection factor (*p* >0.05). Secondly, age might be an important affecting factor. Empirical studies reported that the predilection age of rumination is teenager, and there existed a significant difference on rumination between girls and boys in children and adolescence, while gender difference on rumination in adults was limited in magnitude [41–44]. Nolen-Hoeksema and Girgus stated that girls during adolescence might face more stressful events of an uncontrollable nature and report greater concern about personal appearance, personal safety, personal worth and interpersonal relationships [45]. Our data came from a young undergraduate sample, and these young adults might pay more attention to self-development and self-improvement, therefore, the gender difference on rumination in this young adult sample might be not significant. Thirdly, sample type might be another important factor. In previous studies with significant gender difference on rumination, the samples mainly stemmed from depression patients or patients with depressed mood, for example, Joormann et al. showed that the mean brooding and reflection scores were higher in clinically depressed individuals as compared with healthy controls [46]. Therefore, gender difference might be non-significant in our non-clinical sample for their low rumination levels. Finally, some demographic characteristics (such as education level or race) of sample might affect the comparison on rumination across gender. In previous studies, these demographic variables were not controlled, while which were well controlled in our study with a high-homogeneity sample.

As Nolen said, “Rumination maintains and exacerbates depression by enhancing negative mood-congruent thinking, impairing problem solving and instrumental behavior, and deterring social support” [47]. In our study, there were significant differences on total scores and four sub-scales of CES-D between the low-level rumination group and high-level group, which showed that participants with high-level rumination had more depressive symptoms than those with low-level rumination, supporting the Nolen’s rumination response theory. Previous researches also suggested that rumination could predict the onset and duration of a depressive episode [6, 13, 48], which is needed to confirm in the future by a longitudinal study. Anyway, this study supported the psychometric properties of the Chinese version of RRS-10, which may help to screen high risky individuals of depression, predict the onset and duration of a depressive episode, and guide the clinical treatment or intervention in the early stage of depression in clinical practice.

Several limitations of our study should be acknowledged. Firstly, no interview for screening psychiatric disorder was conducted in this self-report study, while given the large sample size, a proportion of the subjects might have been affected by psychiatric disorder (e.g., mood disorder). Secondly, we just used a convenient sample of university students rather than a clinical sample. Further longitudinal studies should be conducted to investigate the psychometric properties of RRS-10 in clinical samples and in more age groups (e.g, adolescence, late adults), and explore the relationship between rumination and depression deeply.

## Conclusion

The RRS-10 has good psychometric properties, as well as measurement equivalence across gender in Chinese undergraduates, which supports that the RRS-10 can be a good self-report measure for rumination of depressed mood in Chinese undergraduates. Furthermore, by comparing latent mean differences, we provided more robust statistical evidence that there were no significant differences on latent structure of the RRS-10 across gender.

## Additional file

Additional file 1: The factor loadings and inter-factor correlations of RRS-10, and measurement equivalence tests of RRS-10 for the CESD. (DOC 51 kb)

## Abbreviations

CES-D: Center for epidemiological studies depression scale
CFA: Confirmatory factor analysis
CFI: Comparative fit index
CI: Confidence interval
CR: Critical ratio
GFI: Goodness of fit index
IFI: Incremental fit index
MGCFA: Multigroup confirmatory factor analysis
PGFI: Parsimonious goodness of fit index
RMSEA: Root mean square error of approximation
RRS-10: 10-item ruminative response scale
SD: Standard deviation
SE: Standard error
TLI: Tucker–Lewis Index
