# Supplementary material for: Psychometric properties of the 10-item ruminative response scale in Chinese university students
Source: BMC Psychiatry. 2017 Apr 28;17:152. doi: 10.1186/s12888-017-1318-y (PMC5410038; doi:10.1186/s12888-017-1318-y)
Supplement: Additional file 1: — The factor loadings and inter-factor correlations of RRS-10, and measurement equivalence tests of RRS-10 for the CESD. (DOC 51 kb) [file 12888_2017_1318_MOESM1_ESM.doc]

| **TableS1: The factor loadings and inter-factor correlations** | | | | | | | | | | | |
| --- | --- | --- | --- | --- | --- | --- | --- | --- | --- | --- | --- |
|  | RRS1 | RRS2 | RRS3 | RRS4 | RRS5 | RRS6 | RRS7 | RRS8 | RRS9 | RRS10 | Brooding-Reflection |
| factor loadings | 0.21 | 0.58 | 0.49 | 0.40 | 0.49 | 0.60 | 0.66 | 0.46 | 0.67 | 0.43 |  |
| inter-factor correlations |  |  |  |  |  |  |  |  |  |  | 0.81 |

**Appendix 1**

**Appendix 2**

**Measurement equivalence in the 10-item Ruminative Response Scale across high- and low- level of depressive symptom in a large Chinese undergraduate sample**

Firstly, we classified respondents as screen-negative and screen-positive based on the CES-D scores. According to the cut-off score of CES-D, 7414 (94.6%) subjects were classified as screen-negatives, and 423 (5.4%) were screen-positives, respectively. Secondly, we tested the measurement equivalence of RRS-10 across screen-negatives and screen-positives (Table S2). The results showed that the RRS-10 was invariant in Model 2 and Model 3 (i.e. metric equivalence, strong equivalence) for both △CFI ≤ 0.01, while not invariant in Model 4 (strict equivalence). Some previous studies found that it is difficult for strict equivalence to meet criteria generally, just partial equivalence can reaches mostly [1-2]. In all, the RRS-10 was partial equivalence between screen-positives and screen-negatives. And Independent Samples T test showed that screen-positives had higher levels of rumination than screen-negatives (see Table S3).

1. Hox JJ, De Leeuw ED, Zijlmans EA: Measurement equivalence in mixed mode surveys. Frontiers in psychology 2015, 6:87.

2. Stevelink SA, van Brakel WH: The cross-cultural equivalence of participation instruments: a systematic review. Disability and rehabilitation 2013, 35(15):1256-1268.

**TableS2: Fit indexes for measurement equivalence tests of RRS-10 for the CESD**

| Model | χ2 | df | IFI | TLI | CFI | RMSEA | GFI | PGFI | model comparison | △CFI | △χ2 (△df) | *P* Value | RMSEA 90%CI | |
| --- | --- | --- | --- | --- | --- | --- | --- | --- | --- | --- | --- | --- | --- | --- |
|  |  |  |  |  |  |  |  |  |  |  |  |  | LO90 | HI90 |
| Model 1 | 495.815 | 56 | 0.967 | 0.946 | 0.967 | 0.032 | 0.987 | 0.503 |  |  |  |  | 0.032 | 0.030 |
| Model 2 | 515.001 | 66 | 0.966 | 0.953 | 0.966 | 0.030 | 0.987 | 0.592 | 2 vs 1 | 0.001 | 19.186(10) | 0.038 | 0.030 | 0.028 |
| Model 3 | 515.815 | 67 | 0.966 | 0.954 | 0.966 | 0.030 | 0.987 | 0.602 | 3 vs 2 | 0.000 | 0.814(1) | 0.367 | 0.030 | 0.027 |
| Model 4 | 792.806 | 81 | 0.946 | 0.940 | 0.946 | 0.034 | 0.975 | 0.718 | 4 vs 3 | 0.020 | 276.99(14) | 0.000 | 0.034 | 0.032 |
| Note: Model 1 = configural equivalence; Model 2 = metric equivalence;Model 3 = strong equivalence; Model 4 = strict equivalence | | | | | | | | | | | | | | |

| **Table S3. Comparison of RRS-10 between screen-positive and screen-negative subjects based on CES-D.** | | | | | |
| --- | --- | --- | --- | --- | --- |
| Scale | screen-positive  (n=423) | screen-negative  (n=7414) | Mean difference | *P* value | Cohen's *d* |
| RRS-10 | 24.00 ± 4.68 | 21.17 ± 4.53 | 2.83 | 0.00 | 0.61 |
| Brooding | 12.47 ± 2.33 | 10.45 ± 2.74 | 2.02 | 0.00 | 0.79 |
| Reflection | 11.53 ± 2.86 | 10.72 ± 2.93 | 0.81 | 0.00 | 0.28 |
